# Synthetic Opioid and Stimulant Co-Involved Overdose Deaths by Occupation and Industry — United States, 2022

**DOI:** 10.15585/mmwr.mm7410a3

**Published:** 2025-03-27

**Authors:** Eric W. Lundstrom, Alexandria Macmadu, Andrea L. Steege, Matthew Groenewold

**Affiliations:** ^1^Epidemic Intelligence Service, CDC; ^2^Division of Field Studies and Engineering, National Institute for Occupational Safety and Health. CDC; ^3^Department of Epidemiology, Brown University School of Public Health, Providence, Rhode Island.

**Summary**

**What is already known about this topic?**

Overdose deaths involving both synthetic opioids and stimulants have increased sharply in recent years. Although some persons who co-use opioids and stimulants have cited motivations related to functionality and alertness in the workplace, occupational patterns of co-use remain uninvestigated.

**What is added by this report?**

In this exploratory analysis of multiple cause of death data from 2022, occupations and industries with higher percentages of psychostimulant involvement in synthetic opioid overdose deaths tended to be physically demanding, whereas those with higher percentages of cocaine involvement tended to be less so.

**What are the implications for public health practice?**

Employers and other entities seeking to implement work-related substance use and overdose prevention programs might need to tailor their approaches based on potential in psychostimulant or cocaine use within a given occupation or industry.

## Abstract

The proportion of synthetic opioid overdose deaths co-involving stimulants has increased in the United States in recent years. Although persons who use opioids have reported increasing stimulant co-use to maintain workplace productivity and alertness, occupational patterns of co-involvement in fatal overdose have not been systematically investigated. In an exploratory study, data on overdose deaths involving synthetic opioids (e.g., fentanyl) from the 2022 National Vital Statistics System were analyzed to characterize patterns of stimulant co-involvement among U.S. residents aged 15–64 years, stratified by decedents’ usual occupation and industry. Of 69,893 fatal synthetic opioid overdoses, 53.6% involved stimulants. Occupation and industry groups with the highest percentages of synthetic opioid overdose deaths co-involving psychostimulants with abuse potential (psychostimulants) were typically physically demanding (e.g., construction and extraction occupations), whereas categories with highest percentages of cocaine co-involvement were generally less physically strenuous (e.g., business and financial occupations); these patterns might reflect differences in desired drug effects, cost, and geographic availability. Work-related interventions might be useful in preventing the development of substance use disorder by decreasing rates of occupational injuries and workplace stress, connecting workers with substance use disorder to treatment resources, and reducing fatal overdose through harm reduction.

## Introduction

Synthetic opioids (mostly fentanyl) now dominate most U.S. opioid overdose deaths, and the proportion of synthetic opioid–involved overdose deaths that co-involved stimulants, such as psychostimulants with abuse* potential (psychostimulants) or cocaine, more than doubled in the United States between 2018 and 2022 (*1*). Recent qualitative investigations have documented that some persons who use opioids report stimulant co-use to counteract the sedative effects of potent synthetic opioids such as fentanyl, thereby enhancing functionality and alertness in their daily life, particularly in the workplace (*2*,*3*). Understanding occupational differences in the types of stimulants co-involved in synthetic opioid-involved overdose deaths might therefore prove useful for tailoring workplace-oriented overdose prevention efforts (*4*). To this end, National Vital Statistics System (NVSS) data from 2022 were analyzed in an exploratory fashion to examine stimulant co-involvement in synthetic opioid overdoses by decedents’ usual occupation and industry.

## Methods

### Data Source

Mortality data for U.S. residents aged 15–64 years were extracted from the 2022 NVSS mortality multiple cause of death files, which include death certificate data reported from U.S. vital statistics jurisdictions. Synthetic opioid-involved overdose deaths were identified using *International Classification of Diseases, Tenth Revision* (ICD-10) underlying cause of death codes for drug poisoning^†^ and ICD-10 multiple cause of death code T40.4 (poisoning by synthetic opioids other than methadone). Cocaine and psychostimulant involvement were identified using ICD-10 multiple cause of death codes T40.5 and T43.6,^§^ respectively; psychostimulants included substances such as methamphetamine, amphetamine, methylphenidate, and 4-methylenedioxy-methamphetamine (MDMA). Occupation and industry information within the NVSS mortality multiple cause file is based on narrative text fields, which are coded to 2012 CDC Census Occupation and Industry codes through a collaboration with the National Institute for Occupational Safety and Health. Decedents whose occupation or industry were coded as “military” were excluded.

### Data Analysis

Numbers of synthetic opioid-involved overdose deaths and the percentages of synthetic opioid-involved overdoses involving any stimulant (i.e., cocaine, psychostimulants, or both) were stratified by decedents’ usual occupation and industry groupings. The percentages of synthetic opioid-involved overdose deaths involving cocaine or psychostimulants within each occupation and industry category were reported. All analyses were conducted using R statistical software (version 4.4.1; R Foundation). This activity was reviewed by CDC, deemed not research involving human subjects, and was conducted consistent with applicable federal law and CDC policy.^¶^

## Results

### Synthetic Opioid-Involved Overdose Deaths with Psychostimulants or Cocaine Involved

A total of 69,893 synthetic opioid-involved overdose deaths among U.S. residents aged 15–64 years were identified, 53.6% of which also involved either psychostimulants or cocaine (Table). The occupation and industry groups with highest percentages of synthetic opioid overdoses involving either psychostimulants or cocaine (excluding those labeled “other”) were farming, fishing, and forestry (57.5%) and mining (55.9%). Those with the lowest percentages of such overdoses were healthcare practitioners and technical (46.7%) occupations and utilities industries (43.4%).

**TABLE:** Percentage of synthetic opioid–involved overdose deaths* co-involving psychostimulants or cocaine,^†^ by decedent’s usual occupation or industry — National Vital Statistics System, United States, 2022

Characteristic	Synthetic opioid overdoses	Percentage involving psychostimulants or cocaine
**Total**	**69,893**	**53.6%**
**Occupation **		
Farming, fishing, and forestry	508	57.5%
Building and grounds cleaning and maintenance	3,287	55.8%
Construction and extraction	11,831	55.6%
Other-housewife	2,680	54.8%
Arts, design, entertainment, sports, and media	1,106	54.3%
Community and social services	481	54.3%
Installation, maintenance, and repair	2,922	53.5%
Personal care and service	2,051	53.1%
Healthcare support	1,397	53.0%
Legal	178	52.8%
Food preparation and serving-related	5,672	52.3%
Transportation and material moving	7,045	52.3%
Architecture and engineering	380	52.1%
Management	2,558	51.8%
Production	3,454	51.8%
Sales and related	3,930	50.7%
Business and financial	696	50.4%
Life, physical, and social sciences	189	50.3%
Education, training, and library	354	50.0%
Office and administrative	2,758	49.4%
Protective Services	665	48.4%
Computer and mathematical	446	47.8%
Healthcare practitioners and technical	1,001	46.8%
Other–misc (excluding housewife)	14,304	55.9%
**Industry **		
Mining	299	55.9%
Construction	12,797	55.7%
Administrative, support, and waste services	3,402	55.2%
Agriculture, forestry, fishing, and hunting	699	54.9%
Arts, entertainment, and recreation	1,384	54.1%
Accommodation and food services	7,023	52.1%
Healthcare and social assistance	4,057	51.0%
Education services	603	50.6%
Wholesale trade	506	50.6%
Manufacturing	4,914	50.5%
Transportation and warehousing	3,411	50.3%
Management of companies and enterprises	16	50.0%
Retail trade	4,497	49.9%
Information	550	49.6%
Real estate, rental, and leasing	497	48.9%
Professional, scientific, and technical services	1,391	48.4%
Finance and insurance	673	48.0%
Public administration	665	46.0%
Utilities	316	43.4%
Other–Misc, missing	18,410	56.2%
Other services (except public administration)	3,783	56.0%

**Abbreviations:** ICD-10 = *International Classification of Diseases, Tenth Revision*; Misc = miscellaneous.

* Synthetic opioid-involved overdose deaths were identified using ICD-10 underlying cause of death codes for poisoning (X40–X44, X60–X64, X85, and Y10–Y14) and multiple cause of death codes for poisoning by synthetic opioids excluding methadone (T40.4).

^†^ Stimulant co-involvement was identified using ICD-10 multiple cause of death codes T40.5 (poisoning by cocaine) or T43.6 (poisoning by psychostimulants with abuse potential).

### Overdose Deaths and Occupation

**FIGURE 1:**
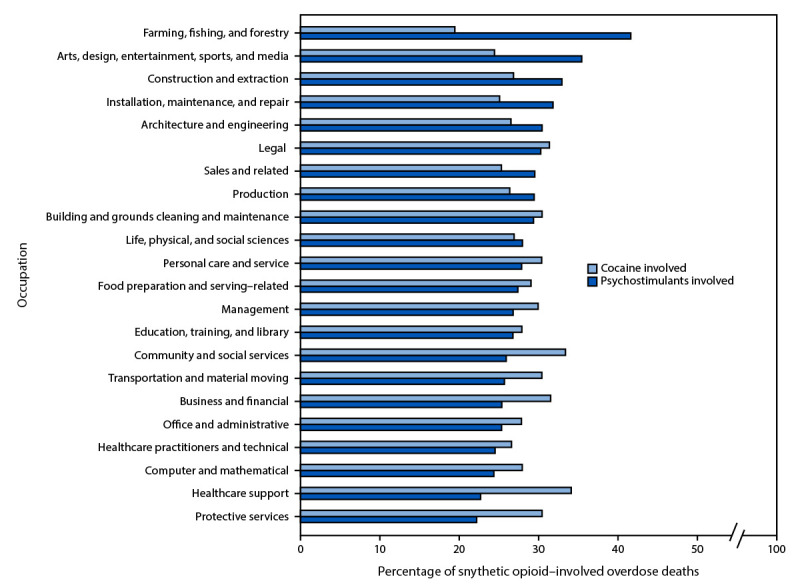
Percentage of fatal synthetic opioid–involved overdoses* co-involving psychostimulants^†^ or cocaine^§^ for 22 major occupation groups^¶^ — National Vital Statistics System, United States, 2022** **Abbreviations:** ICD-10 = *International Classification of Diseases, Tenth Revision*; Misc = miscellaneous. * Synthetic opioid–involved overdose deaths were identified using ICD-10 underlying cause of death codes for poisoning (X40–X44, X60–X64, X85, and Y10–Y14) and multiple cause of death codes for poisoning by synthetic opioids excluding methadone (T40.4). ^†^ Psychostimulant involvement was identified using ICD-10 multiple cause of death code T43.6, poisoning by psychostimulants with abuse potential, which includes poisoning by substances such as methamphetamine, amphetamine, methylphenidate, and 4-methylenedioxy-methamphetamine and excludes poisoning by cocaine. ^§^ Cocaine involvement was identified using ICD-10 multiple cause of death code T40.5, poisoning by cocaine. ^¶^ Excluding “other–Misc, missing” or “Other-Housewife” occupations. ** ICD-10 multiple cause of death codes are not mutually exclusive. Therefore, decedents included in this analysis might have had both psychostimulants and cocaine present at the time of their death and the percentages included in this figure might not equal the total percent with any stimulant present.

Occupations with the highest percentages of synthetic opioid-involved overdoses involving psychostimulants (excluding those labeled “other”) were farming, fishing, and forestry (41.7%); arts, design, entertainment, sports, and media (35.5%); construction and extraction (33.0%); installation, maintenance, and repair (31.9%); and architecture and engineering (30.5%) (Figure 1). Occupations with the highest percentages of synthetic opioid-involved overdoses involving cocaine were healthcare support (34.2%); community and social services (33.5%); business and financial (31.6%); legal (31.5%); and protective services (30.5%).

### Overdose Deaths and Industry

**FIGURE 2:**
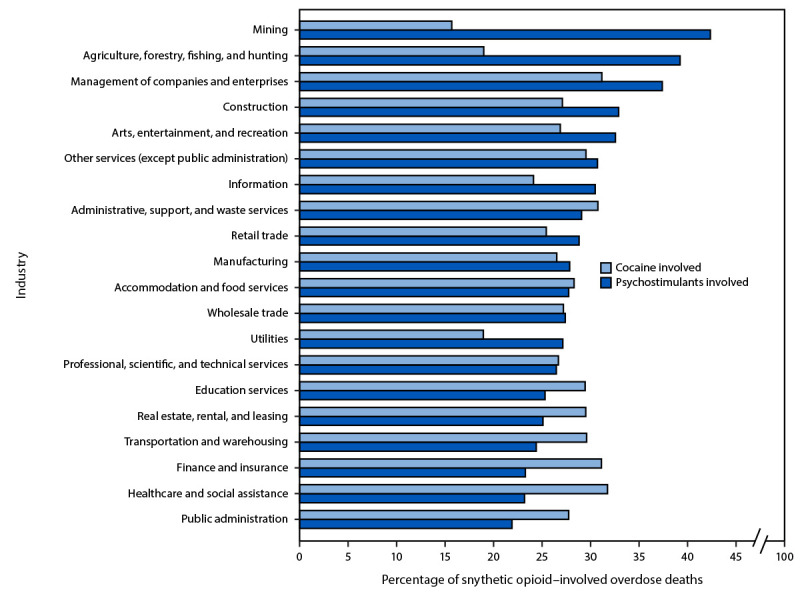
Percentage of fatal synthetic opioid–involved overdoses* co-involving psychostimulants^†^ or cocaine^§^ for 20 major industry groups^¶^ — National Vital Statistics System, United States, 2022** **Abbreviations:** ICD-10 = *International Classification of Diseases, Tenth Revision*; Misc = miscellaneous. * Synthetic opioid–involved overdose deaths were identified using ICD-10 underlying cause of death codes for poisoning (X40–X44, X60–X64, X85, and Y10–Y14) and multiple cause of death codes for poisoning by synthetic opioids excluding methadone (T40.4). ^†^ Psychostimulant involvement was identified using ICD-10 multiple cause of death code T43.6, poisoning by psychostimulants with abuse potential, which includes poisoning by substances such as methamphetamine, amphetamine, methylphenidate, and 4-methylenedioxy-methamphetamine and excludes poisoning by cocaine. ^§^ Cocaine involvement was identified using ICD-10 multiple cause of death code T40.5, poisoning by cocaine. ^¶^ Excluding “other–Misc, missing” industries. ** ICD-10 multiple cause of death codes are not mutually exclusive. Therefore, decedents included in this analysis might have had both psychostimulants and cocaine present at the time of their death, and the percentages included in this figure might not equal the total percentage with any stimulant present.

Industries with the highest percentages of synthetic opioid overdose deaths involving psychostimulants were mining (42.5%); agriculture, forestry, fishing, and hunting (39.3%); management of companies and enterprises (37.5%); construction (33.0%); and arts, entertainment, and recreation (32.7%) (Figure 2). Industries with the highest percentages of such deaths with cocaine involvement included healthcare and social assistance (31.8%); management of companies and enterprises (31.3%); finance and insurance (31.2%); real estate, rental, and leasing (30.4%); administrative, support, and waste services (30.8%); education services (29.5%); and transportation and warehousing (29.7%).

## Discussion

In this exploratory analysis of occupational patterns of psychostimulant or cocaine involvement in synthetic opioid-involved overdose deaths, occupations and industries with high percentages of synthetic opioid-involved overdoses co-involving psychostimulants tended to be those likely involving manual labor. Occupations and industries with higher percentages of cocaine involvement were those often considered to be less physically strenuous.

One potential explanation for these patterns is that psychostimulants are better suited for counteracting opioid-involved lethargy in physically demanding occupations, particularly given that the drug effects of psychostimulants such as methamphetamine are longer-lasting than those of cocaine (*5*). Previous studies have observed that persons who use opioids report intentional co-use of methamphetamine to improve functioning in their fast-paced, manual occupations (*2*). Similarly, workers in construction and landscaping jobs report using methamphetamine to reduce pain associated with working in these labor-intensive jobs (*6*). Another contributing factor could be the relative difference in cost between psychostimulants and cocaine. Persons who work in more physically demanding occupations (e.g., farming, fishing, and forestry occupations) are frequently paid lower wages than are those in occupations requiring less manual labor (e.g., business and financial occupations) (*7*); use of psychostimulants such as methamphetamine might therefore be more prevalent within certain occupation groups given that it tends to be less expensive than cocaine (*3*,*8*).

Geography might also play a role in the overlap between employment in certain industries and co-use of opioids with specific stimulants. For instance, data indicate that recent increases in psychostimulant-involved opioid overdose deaths were most rapid in the Appalachian region (*9*). Compared with other regions, Appalachia has higher rates of employment in the mining industry (*10*), which had the highest percentage of psychostimulant involvement of any industry in this study. Conversely, industries with high cocaine involvement, such as education services or healthcare and social assistance, might be less geographically localized. Others, such as financial services, might be more common in urban areas, where rates of cocaine-involved overdoses are higher than are those in rural areas (*1*).

### Limitations

The findings in this report are subject to at least five limitations. First, although qualitative reports suggest that many persons who co-use opioids and stimulants do so intentionally, particularly in the context of counteracting opioid-related lethargy in the workplace, NVSS does not include data on intent of co-use. Second, usual (or longest held) occupation and industry within NVSS are collected as part of death certificate reporting; for some decedents, there might be a discrepancy between what occupation or industry they were usually employed in and that in which they were employed at the time of death. Third, this report focused on stimulant involvement in synthetic opioid-involved overdose deaths, and its findings cannot be generalized to opioid overdoses not involving synthetic opioids. However, as of 2023, synthetic opioids other than methadone were involved in approximately 70% of overdose fatalities in the U.S. and were therefore the focus of this report (*1*). Fourth, within-group differences in proportions in stimulant co-involved deaths tended to be small. Finally, this analysis was exploratory, with no guiding hypotheses; therefore, these findings should be considered hypothesis-generating and warrant confirmation.

### Implications for Public Health Practice

These hypothesis-generating findings warrant confirmation but point to a potential role for work-related substance use and overdose prevention interventions. The National Institute for Occupational Safety and Health has developed the Workplace Supported Recovery (WSR) initiative, which guides employers in bolstering the employment and retention of persons with substance use disorders and in facilitating access to treatment (*4*). The WSR initiative also aims to address the determinants of substance use disorders and overdose through the reduction of work-related risk factors, including occupational injury and work-related stress (*4*). To maximize their potential benefit, WSR and other workplace-oriented interventions might need to tailor their approaches based on potential psychostimulant or cocaine use within a given occupation or industry. Nevertheless, increased access to harm reduction resources and evidence-based treatments for opioid use disorder and stimulant use disorder, both within and outside of a workplace setting, will be needed to address the current U.S. overdose crisis.

## Footnotes

* The term “abuse” is used in this report to maintain consistency with *International Classification of Diseases, Tenth Revision* (ICD-10) multiple cause of death coding and previous publications from CDC describing stimulant-involved overdose deaths. However, the term should otherwise be limited in use, because "abuse" connotes violent and deliberately harmful behavior and reinforces stigmatizing attitudes toward people who use drugs, thereby undermining efforts to engage this population. A list of preferred terms to use in the context of substance use is available online. https://nida.nih.gov/nidamed-medical-health-professionals/health-professions-education/words-matter-terms-to-use-avoid-when-talking-about-addiction

† ICD-10 underlying cause of death codes X40–X44, X60–X64, X85, and Y10–Y14.

§ ICD-10 multiple cause of death code T43.6, poisoning by psychostimulants with abuse potential, includes poisoning by substances such as methamphetamine, amphetamine, methylphenidate, and MDMA and excludes poisoning by cocaine.

¶ 45 C.F.R. part 46.102(l)(2), 21 C.F.R. part 56; 42 U.S.C. Sect. 241(d); 5 U.S.C. Sect. 552a; 44 U.S.C. Sect. 3501 et seq.

## References

[1] CDC. National Center for Health Statistics mortality data on CDC WONDER. Atlanta, GA: US Department of Health and Human Services, CDC; 2023 Accessed May 24, 2024. https://wonder.cdc.gov/mcd.html

[2] Baker R, Leichtling G, Hildebran C, “Like yin and yang”: perceptions of methamphetamine benefits and consequences among people who use opioids in rural communities. J Addict Med 2021;15:34–9. 10.1097/ADM.0000000000000669 32530888 PMC7734765

[3] Fredericksen RJ, Baker R, Sibley A, Motivation and context of concurrent stimulant and opioid use among persons who use drugs in the rural United States: a multi-site qualitative inquiry. Harm Reduct J 2024;21:74. 10.1186/s12954-024-00986-z 38561753 PMC10985853

[4] Osborne JC, Chosewood LC. NIOSH responds to the U.S. drug overdose epidemic. New Solut 2021;31:307–14. 10.1177/10482911211040754 34431384 PMC10170552

[5] National Institute on Drug Abuse. How is methamphetamine different from other stimulants, such as cocaine? Bethesda, MD: US Department of Health and Human Services, National Institutes of Health, National Institute on Drug Abuse; 2020. https://nida.nih.gov/sites/default/files/methrrs.pdf

[6] Hansen ER, Carvalho S, McDonald M, Havens JR. A qualitative examination of recent increases in methamphetamine use in a cohort of rural people who use drugs. Drug Alcohol Depend 2021;229(Pt B):109145. 10.1016/j.drugalcdep.2021.109145 34763138 PMC8665094

[7] US Bureau of Labor Statistics. Occupational employment and wage statistics. Washington, DC: US Bureau of Labor Statistics; 2023. https://www.bls.gov/oes/current/oes_nat.htm

[8] Mansoor M, McNeil R, Fleming T, Characterizing stimulant overdose: a qualitative study on perceptions and experiences of “overamping”. Int J Drug Policy 2022;102:103592. 10.1016/j.drugpo.2022.103592 35114520 PMC9381030

[9] Kline D, Bunting AM, Hepler SA, Rivera-Aguirre A, Krawczyk N, Cerda M. State-level history of overdose deaths involving stimulants in the United States, 1999–2020. Am J Public Health 2023;113:991–9. 10.2105/AJPH.2023.307337 37556789 PMC10413741

[10] US Energy Information Administration. Annual Coal Report Table 18. average number of employees by state and mine type, 2022 and 2021. Washington, DC: US Energy Information Administration, US Department of Energy; 2022. https://www.eia.gov/coal/annual/pdf/table18.pdf

